# Land Use/Cover Change in the Middle Reaches of the Heihe River Basin over 2000-2011 and Its Implications for Sustainable Water Resource Management

**DOI:** 10.1371/journal.pone.0128960

**Published:** 2015-06-26

**Authors:** Xiaoli Hu, Ling Lu, Xin Li, Jianhua Wang, Ming Guo

**Affiliations:** Cold and Arid Regions Environmental and Engineering Research Institute, Chinese Academy of Sciences, 320 Donggang West Road, Lanzhou, 730000, China; Beijing Normal University, CHINA

## Abstract

The Heihe River Basin (HRB) is a typical arid inland river basin in northwestern China. From the 1960s to the 1990s, the downstream flow in the HRB declined as a result of large, artificial changes in the distribution of water and land and a lack of effective water resource management. Consequently, the ecosystems of the lower reaches of the basin substantially deteriorated. To restore these degraded ecosystems, the Ecological Water Diversion Project (EWDP) was initiated by the Chinese government in 2000. The project led to agricultural and ecological changes in the middle reaches of the basin. In this study, we present three datasets of land use/cover in the middle reaches of the HRB derived from Landsat TM/ETM+ images in 2000, 2007 and 2011. We used these data to investigate changes in land use/cover between 2000 and 2011 and the implications for sustainable water resource management. The results show that the most significant land use/cover change in the middle reaches of the HRB was the continuous expansion of farmland for economic interests. From 2000 to 2011, the farmland area increased by 12.01%. The farmland expansion increased the water resource stress; thus, groundwater was over-extracted and the ecosystem was degraded in particular areas. Both consequences are negative and potentially threaten the sustainability of the middle reaches of the HRB and the entire river basin. Local governments should therefore improve the management of water resources, particularly groundwater management, and should strictly control farmland reclamation. Then, water resources could be ecologically and socioeconomically sustained, and the balance between upstream and downstream water demands could be ensured. The results of this study can also serve as a reference for the sustainable management of water resources in other arid inland river basins.

## Introduction

Land use/cover is significant to key land surface process [1–2] by significantly impacting regional climate change [3], water resource availability [4–5], and biodiversity loss and soil degradation [6]. Land use/cover is therefore a major consideration for sustainable development and is emerging as a core issue in global environmental change and sustainable development [7–8].

Changes in land use/cover have occurred on a global scale in recent decades, with the conversion of forests to cropland and the ongoing conversion of cropland to urban areas [6]. The rapid expansion of farmland is associated with a large increase in the quantity of water used for irrigation, which can be linked to a decrease in the amount of water available to ecosystems in nearly all arid areas [9]. At the same time, industrial and domestic water demands have increased rapidly as a result of increasing economic development and urbanization. Thus, water resource utilization has approached or exceeded the threshold of natural water resources in some arid inland basins [10]. The exploitation of water resources has resulted in devastating environmental and ecological disasters in downstream areas and throughout the entire river basin [11].

The Heihe River Basin (HRB) is a typical inland river basin in the arid region of northwestern China. The river originates in the Qilian Mountains in Qinghai Province, flows through the Hexi Corridor of Gansu Province, and then enters the Alxa Plateau of the Inner Mongolia Autonomous District. The water from the Heihe River is important to agricultural production and ecosystem stabilization in the middle and lower reaches of the HRB and is highly sensitive to climate change and human activities in the area [12–15]. The climate in the HRB has remained in a relatively stable warm-wet state since the 1960s and has not experienced strong changes due to global warming [15–18]. Human activities have therefore become the dominant driver of changes in the water resources of the HRB over the last 60 years. With large-scale developments in the use of both water and land resources, human-induced water shortages have become a serious environmental, economic and social problem [19–22]. Water consumption increased dramatically between the 1960s and the 1990s due to a continuous increase in the population and the expansion of farmland in the middle reaches of the HRB. This increased consumption resulted in a sharp decline in the amount of water in the lower reaches of the basin. In the 1950s, the annual runoff into the lower reaches was approximately 12×10^8^ m^3^ year^-1^, but in the 1990s, the runoff was less than 7×10^8^ m^3^ year^-1^ [23–24]. Consequently, a large area of *Populus euphratica* forest died, and the plant coverage in the area dramatically declined [25]. Gaxun Nuur Lake dried up in 1961, and Sogo Nuur Lake dried up in 1992 [26]. To prevent further ecosystem deterioration and to restore the environment in the lower reaches, the Chinese Government implemented the Ecological Water Diversion Project (EWDP) in 2000. According to this project, the middle reaches must transfer 9.5×10^8^ m^3^ of water to the lower reaches in ‘normal’ years (a ‘normal’ year is defined as a year in which the upper reaches discharge 15.8 × 10^8^ m^3^ of water). Additionally, to achieve this water reallocation goal and to alleviate the conflicts between upstream and downstream consumption demands, the implementation of particular water conservation and ecological protection policies was also recommended within the framework of the EWDP. These policies include the Water Conservation Society, ecological migration, the ‘grain-for-green’ policy (referred to as the Conversion of Farmland to Forests and Grassland Program) [27–28], and the Wetland Conservation Project. The implementation of these policies has substantially restored the environment in the lower reaches of the basin. For example, the inland lake has reappeared, and the vegetation coverage has started to increase [29–30].

The use of surface water resources in the middle reaches of the HRB was restricted as part of the EWDP because more stream flow was reallocated for the downstream areas. This restriction has impacted the income of local farmers and has affected environmental and social stability in the region [31–32]. Competition between supply and demand for water in the middle reaches of the basin has intensified over the last ten years as land resources have continued to be exploited. An updated land use/cover dataset is therefore necessary to quantitatively evaluate the relationships between land use/cover, water resources and sustainable ecological-economic-social development. The most recent studies on land use/cover in the middle reaches of the HRB are based on a dataset for 1960–2005 [31, 33–36]; thus, data for recent years are not included. More importantly, there is no clear and definitive assessment of the relationships between land use/cover and water resources, particularly under the strict water-land conditions implemented by the EWDP. The research and monitoring of changes in land use/cover in the middle reaches of the HRB after the implementation of the EWDP are therefore critical to the planning and management of water resources and the sustainable development of the HRB.

The aim of this study is to supply updated, accurate and reliable information on changes in land use/cover between 2000 and 2011. A further aim is to investigate the impact of land use/cover on sustainable water resources management in the region. The allocation of water resources for upstream, midstream and downstream reaches in inland river regions is an active international field of study and topic of debate [9, 37]. For example, similar problems have been investigated in the Aral Sea Basin in Central Asia [38–40] and in the Tarim River Basin in northwestern China [41]. We therefore hope that the present study will serve as a reference for the management of water resources in these regions.

## Study area

**Fig 1:**
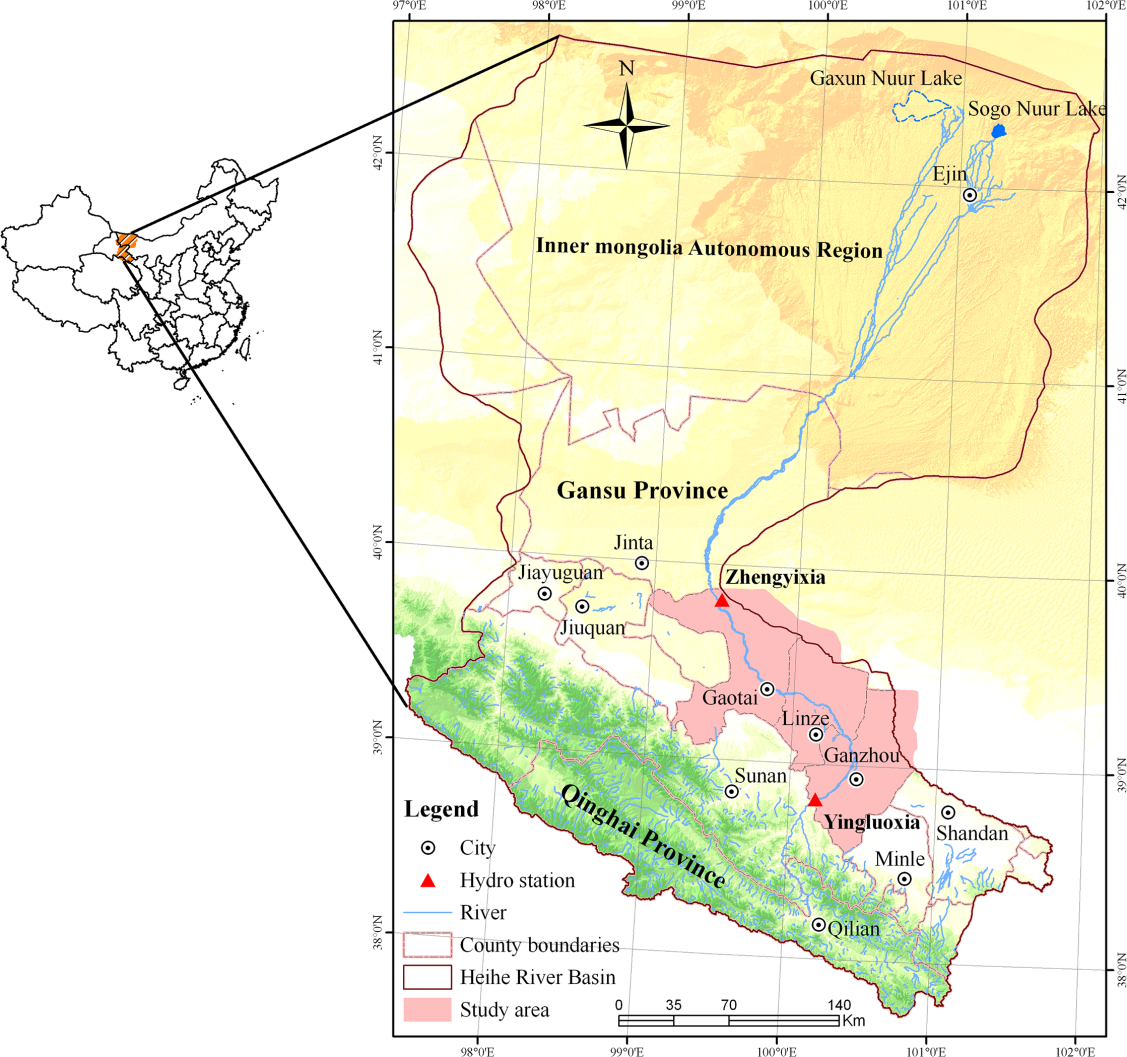
Location of the study area.

The HRB is located between 97.1°-102.0°E and 37.7°-42.7°N, which covers a total area of 143,000 km^2^ (Fig 1). Based on the locations of two key hydrological stations along the main branch of the Heihe River, the Yingluoxia and Zhengyixia stations, the entire river basin can be divided into three sections. The upper reaches are located upstream of the Yingluoxia hydrological station and represent the water resource formation area of the Heihe River. The middle reaches are located between the two hydrological stations and are a major consumer of water resources. The lower reaches are located downstream of the Zhengyixia hydrological station. In this region, the natural oases are dominated by grasslands, which are relatively small, fragmented and easily disturbed. Our study focused on the middle reaches of the HRB. The study area includes the Ganzhou District, Linze County and Gaotai County (10,685 km^2^).

The study area is an irrigated agricultural district, typical of many in the HRB. The annual average air temperature is 6–8°C, the annual precipitation is approximately 150 mm, and the annual potential evaporation is 1000–2000 mm. The Heihe River water flowing past the Yingluoxia hydrological station is the primary surface water resource in the study area. The use of surface water in the study area is high and dominated by irrigation, which accounts for 90% of the total water consumption in the area [42].

## Materials and Methods

### Land use/cover data

The analysis of land use/cover changes in the middle reaches of the HRB was based on three land use/cover datasets, i.e., 2000, 2007 and 2011. The EWDP was formally implemented in 2000; hence, we chose this year as the baseline for our analysis. In 2008, the middle reaches of the HRB were subjected to major policy changes in the form of the Wetland Conservation Project, which was implemented by the local government to restore wetlands along the Heihe River and to protect existing wetlands from being converted into farmland or built-up land. Thus, we chose 2007 as an important transition point for land use change. We chose 2011 to evaluate the spatial-temporal dynamics of land use/cover over the 12 years following the implementation of the EWDP.

These land use/cover data were derived from 30-m-resolution Landsat TM/ETM+ images. The Landsat TM/ETM+ images were acquired in the three study years (2000, 2007 and 2011) over the entire study area. The path/row numbers were 133/033 and 134/033 (Table 1). These images were downloaded from the U.S. Geological Survey (USGS) (http://glovis.usgs.gov/). In addition, 2.5-m-resolution Satellite Pour l’Observation de la Terre (SPOT) images were also used for an in-depth investigation into land use/cover changes and for validation in typical regions. The high-resolution SPOT images were acquired on March 29, 2008, and July 4, 2008, for Ganzhou District and Linze County, respectively.

**Table 1:** The TM/ETM+ data used in this study.

NO.	Acquisition date	Path-row number	Sensor
1	2000-08-10	133/033	ETM+
2	2000-06-14	134/033	ETM+
3	2007-08-22	133/033	TM
4	2007-08-13	134/033	TM
5	2011-08-01	133/033	TM
6	2011-08-08	134/033	TM

Each of these images was rectified with an Albers Equal-Area Conic Projection based on topographical maps at a scale of 1:50,000. The SPOT images were also projected onto the same framework (Albers, WGS84 datum) using Google Earth images for reference. For geometric rectification, 20–30 control points at uniformly distributed locations were selected in each image. The root-mean-squared errors (RMS) were less than one pixel.

The land use/cover data in 2000 were obtained from the National Land Use/cover Database of China (NLUD-C) at a scale of 1:100,000, which was compiled by Liu [42–43] via visual interpretation. In the visual interpretation method, interpreters first analyze and interpret remote sensing images on a computer based on their experience and knowledge and then interactively draw boundaries and label attributes for every polygon of different land use/cover type using GIS software [43–44]. This method can ensure the consistency and accuracy of the data processing [43–44]. The land use/cover data in 2007 and 2011 were updated by visual interpretation based on the land use/cover data in 2000 and Landsat TM/ETM+ images in 2000, 2007 and 2011. Following the NLUD-C classification system [43] and land use characteristics in the HRB, a two-tier hierarchical classification system was applied. This classification system comprehensively considered the characteristics of land use and land cover in the HRB and included seven primary land use/cover types: farmland, forestland, grassland, water body, built-up land, wetland and desert. The farmland type was subdivided based on cultivation conditions; the forestland and grassland types were subdivided based on vegetation coverage; and the water body type was subdivided to emphasize the difference between natural and anthropogenic attributes. Descriptions of these land use/cover types are presented in Table 2 [43–44].

**Table 2:** Description of the land use/cover classification system used in this study.

Level 1 type	Level 2 type	Description
Farmland	Hilly dryland	Rain-fed farmland without irrigation
	Plain dryland	Farmland with guaranteed water source or irrigation facilities
Forestland	Arboreal forest	Natural or plantation forest with a canopy cover >30%
	Shrub forest	Woodland or shrub with a canopy cover >40% and a height ≤ 2 m
	Sparse forest	Natural or plantation forest with a canopy cover ≤30%
	Other forest	Economic woodland, such as orchards and nurseries
Grassland	Thick grassland	Natural or artificial grassland with a canopy cover >50%
	Moderate grassland	Natural or artificial grassland with a canopy cover between 20% and 50%
	Sparse grassland	Natural or artificial grassland with a canopy cover ≤20%
Water body	River and canal	Land covered by rivers, including canals
	Lake	Land covered by lakes
	Reservoir and pond	Man-made facilities for water reservation
	Overflow land	Land between normal water level and flood level
Built-up land		Residences, transportation networks, and other building structures, including land for urban occupation
Wetland		Land area whose soil is saturated with moisture either permanently or seasonally
Desert		Land yet to be utilized, including land deemed difficult to use, such as sandy land, saline, barren soil, and bare rock

The land use/cover data in 2000 were verified by Liu, and the interpretation accuracy for the land use/cover classification was greater than 90% [43–44]. We used high-resolution SPOT images in place of ground-truth information to estimate the accuracy of the land use/cover classification in 2007 as follows. First, high-resolution SPOT images for two typical regions (the northern area of Ganzhou District and Pingchuan in Linze County) were manually interpreted. Second, the 2.5-m-resolution interpreted results of the SPOT images were resampled to a resolution of 30 m to approximate the ground-truth data from the TM/ETM+ images [45–46]. Finally, a superposition analysis was conducted on the interpreted results of the TM/ETM+ and SPOT images, and a confusion matrix was established. The validation results produced a classification accuracy of 82.96% and a Kappa statistic of 0.731 in the northern area of Ganzhou District. In Pingchuan in Linze County, the classification accuracy was 92.40%, and the Kappa statistic was 0.854. To verify the accuracy of the land use/cover in 2011, a field investigation was conducted in September 2012. A GPS system was used to obtain the locations of ground-truth observations in the study area. A total of 120 verification points and approximately 280 photographs were obtained during the field campaign. The validation results indicated that the overall accuracy of the land use/cover classification in 2011 approached 94.4%. The relevant data will be added to WestDC (http://www.heihedata.org/).

### Hydrological data

The hydrological data for this study included annual runoff and annual groundwater level data. The annual runoff data were computed from monthly records at two stations, Yingluoxia and Zhengyixia, which separately record runoff at the outlet of the mountainous area of the upper reaches and the water flow reaching the lower reaches of the basin. The instrumental records span 1990–2011. The annual groundwater-level data were computed from monthly records at 42 observation wells of the Heihe River Bureau and the Environmental and Ecological Science Data Center for West China (http://westdc.westgis.ac.cn/) [47].

### Other data

Socioeconomic data were collected from the Zhangye Statistical Yearbook. These socioeconomic data are time series of the entire study period from 2000 to 2011. Information on national policy was obtained from local governments and from related literature. This information includes various policies implemented during the study period, such as the so-called ‘grain-for-green’ policy, the Wetland Conservation Project and the Water Conservation Society. These data were used to analyze and identify the driving forces of land use/cover changes in the middle reaches of the HRB.

### Land use/cover change detection

Three land use/cover datasets were statistically analyzed to identify changes in various land use/cover types during the study period. In addition, a transition matrix was produced using the ArcGIS software. Quantitative area data for the overall land use/cover changes, as well as increases and decreases in the spatial extent of each cover type, between 2000 and 2011 were compiled. The transition matrix provides information on the magnitude and direction of land use changes in the study area.

## Results and Analysis

### Distribution of various land use/cover types

**Fig 2:**
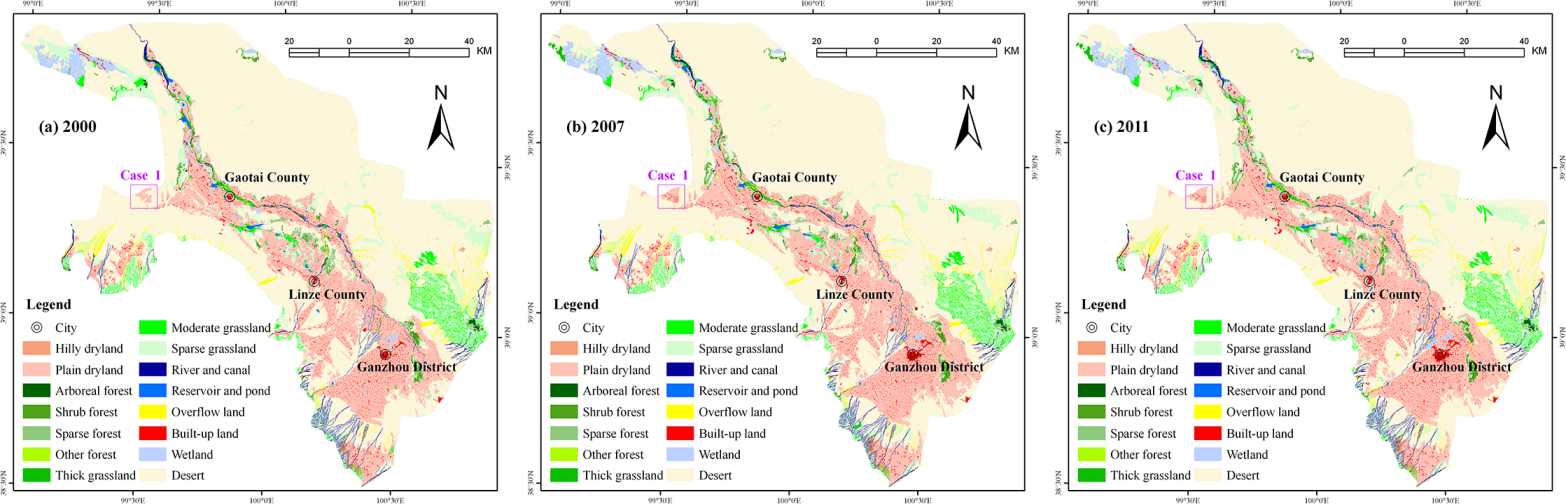
Maps of land use/cover in the study area over 2000–2011 (case 1 was a typical example of land use/cover change driven by migrants).

Desert, farmland and grassland are the largest and most widely distributed types of land use/cover in the middle reaches of the HRB (Fig 2). Desert is mainly located in the piedmont region of the study area. Farmland is mainly located along a corridor that follows the Heihe River and on either side of irrigation canals. Grassland is mainly located in the northern mountains, hills, within the transition zone between the mountains and plains, and along oasis edges. Forestland is mainly located in the transition zone between oasis and desert, in overflow land, and in areas of desert surrounded by oases. Built-up land in the countryside is dominated by residential areas and is located on both sides of roads and on both sides of the river. Wetlands are mainly found in the suburbs of Ganzhou District and in Yanchi, Gaotai County.

### Land use/cover changes from 2000 to 2011

Considerable changes occurred in land use/cover in the middle reaches of the HRB from 2000 to 2011, as shown in Table 3. The area covered by farmland and built-up land greatly increased. The total area of farmland increased from 2132.45 km^2^ in 2000 to 2388.59 km^2^ in 2011, an increase of 256.14 km^2^ or 12.01% of the total farmland area in 2000. The total built-up land area increased by 31.46 km^2^, or 23.96% of the total built-up land area in 2000. The total forestland area increased by 7.17%. In contrast, areas covered by grasslands, water bodies and deserts decreased continuously from 2000 to 2011. The total grassland area decreased by 69.24 km^2^, with a particularly dramatic decrease in the sparse grassland area. The total area covered by water bodies decreased by 20.87 km^2^, which is equivalent to 6.36% of the total water body area in 2000. The desert area decreased by approximately 3.08%. The spatial extent of wetlands only changed slightly from 2000 to 2011 (0.63%). However, wetland areas exhibited two trends from 2000 to 2011: a decrease of 5.78% from 2000 to 2007, followed by an increase of 5.46% from 2007 to 2011.

**Table 3:** Changes in the primary types of land use/cover.

Year	Parameter	Farmland	Forestland	Grassland	Water body	Built-up land	Wetland	Desert
**2000**	**Area (km** ^**2**^ **)**	2132.45	134.34	1106.77	328.02	131.27	157.47	6694.70
	**Percent (%)**	19.96	1.26	10.36	3.07	1.23	1.47	62.66
**2007**	**Area (km** ^**2**^ **)**	2340.79	135.93	1042.78	309.65	154.32	148.38	6553.17
	**Percent (%)**	21.91	1.27	9.76	2.90	1.44	1.39	61.33
**2011**	**Area (km** ^**2**^ **)**	2388.59	143.97	1037.52	307.14	162.73	156.48	6488.58
	**Percent (%)**	22.35	1.35	9.71	2.87	1.52	1.46	60.73
**2000–2007**	**Area change (km** ^**2**^ **)**	208.34	1.58	-63.99	-18.36	23.04	-9.09	-141.54
	**Percent (%)**	9.77	1.18	-5.78	-5.60	17.55	-5.78	-2.11
**2007–2011**	**Area change (km** ^**2**^ **)**	47.81	8.04	-5.26	-2.51	8.41	8.10	-64.58
	**Percent (%)**	2.04	5.92	-0.50	-0.81	5.45	5.46	-0.99
**2000–2011**	**Area change (km** ^**2**^ **)**	256.14	9.63	-69.24	-20.87	31.46	-0.99	-206.12
	**Percent (%)**	12.01	7.17	-6.26	-6.36	23.96	-0.63	-3.08

### Transition matrix analysis

The transition matrix for land use/cover types between 2000 and 2011 is shown in Table 4. The increase in the farmland area is mainly attributed to a decrease in desert and grassland areas. Approximately 181.11 km^2^ of deserts and 71.51 km^2^ of grasslands were converted into farmland. The newly reclaimed farmland was mainly located in the transition zone between the oasis and desert. Observations from the field campaign also confirmed that farmland reclamation was substantial in the middle reaches of the HRB.

**Table 4:** The dynamic transition matrix of land use/cover types between 2000 and 2011 (km^2^).

2000	2011						
	Farmland	Forestland	Grassland	Water body	Built-up land	Wetland	Desert
**Farmland**	2082.40	6.00	5.71	2.13	24.32	6.12	5.77
**Forestland**	26.88	100.40	2.61	0.53	0.13	0.27	3.52
**Grassland**	71.51	16.28	907.86	2.62	0.81	4.69	103.00
**Water body**	12.03	2.09	8.42	298.93	0.07	6.16	0.33
**Built-up land**	0.00	0.02	0.00	0.00	131.25	0.00	0.00
**Wetland**	14.67	0.75	8.39	0.80	0.56	132.13	0.17
**Desert**	181.11	18.44	104.54	2.13	5.59	7.10	6375.79

The total area of built-up land increased significantly over the study period. The expansion in built-up land mainly occurred at the expense of farmland; approximately 24.32 km^2^ of farmland was converted into built-up land. The increase in the built-up land area was greatest in Ganzhou District.

Areas of forestland increased between 2000 and 2011, primarily because of the conversion of deserts and grasslands. During 2000 and 2011, the total areas of desert and grassland that were converted to forestland were 18.44 km^2^ and 16.28 km^2^, respectively.

The total area of grassland decreased throughout the study period as grasslands were converted to farmland and desert. Between 2000 and 2011, approximately 71.51 km^2^ of grassland was converted to farmland. The conversion of grassland to desert mainly occurred between 2000 and 2007 as water resources became scarcer.

The spatial extent of water bodies decreased between 2000 and 2011. Approximately 12.03 km^2^ of water bodies was transformed into farmland and 8.42 km^2^ was transformed into grassland over the study period. The primary causes of the reduced area of water bodies were the conversion of overflow land to farmland and grassland and the reduction in surface water resources following the EWDP implemented in 2000.

Because of the impact of the EWDP, the wetland area decreased at the start of the study period and then increased. Between 2000 and 2007, wetland areas decreased by 9.09 km^2^; however, between 2007 and 2011, wetland areas increased by 8.10 km^2^ (Table 3). Between 2000 and 2007, a large wetland area was reclaimed for farmland (approximately 13.16 km^2^), and surface water resources decreased. The subsequent increase in the wetland area can be explained by the implementation of the Wetland Conservation Project in 2008 within the framework of the EWDP, which resulted in an overall increase in wetland areas between 2007 and 2011.

## Discussion

The above analysis reveals that farmland significantly expanded between 2000 and 2011. Why did farmland substantially increase after the implementation of the EWDP in the HRB in 2000? Was the increase in the farmland due to a significant increase in water resources, the implications of a national policy or economic factors?

### Water resource availability vs. land use/cover change

**Fig 3:**
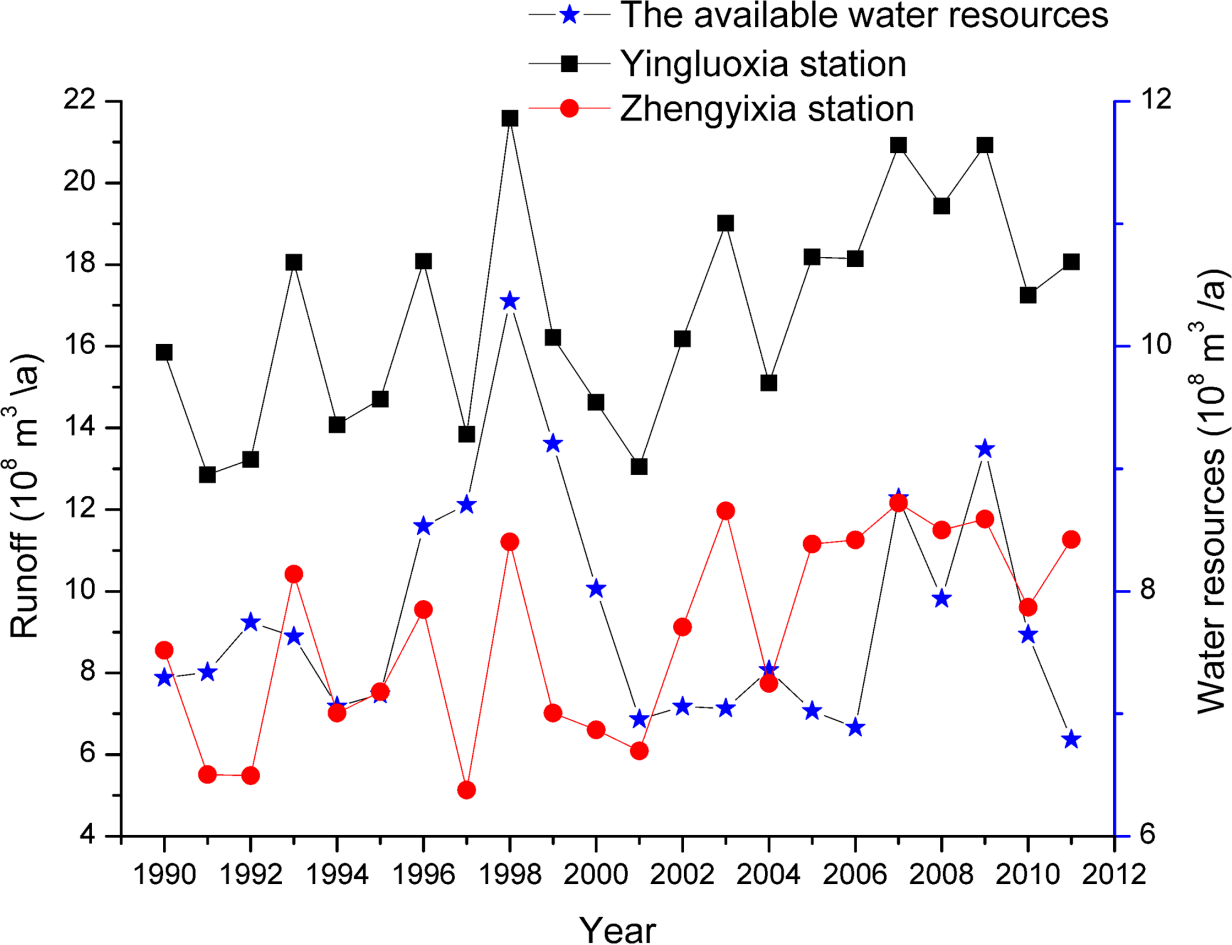
Run-off flows released between the Yingluoxia and Zhengyixia hydrological stations and the available surface water resources in the middle reaches of the HRB from 1990 to 2011.

To address these questions, the relationships between water resource availability and land use/cover change must be analyzed. We first analyzed the runoff measured at the Yingluoxia hydrological station. Between 1945 and 2000, the mean annual runoff observed at the Yingluoxia hydrological station was 16.08 × 10^8^ m^3^. However, the mean annual runoff observed between 2000 and 2011 was 17.57 × 10^8^ m^3^, which represents an increase of 9.3% compared with 1945–2000. Only three years between 2000 and 2011 (2000, 2001 and 2004) corresponded to a total runoff of less than 16.08 × 10^8^ m^3^, whereas the annual runoff for all other years exceeded 16.08×10^8^ m^3^ (Fig 3). These data suggest that the average runoff from the Heihe River has been greater than the ‘normal’ level since 2000 and has been continuously high since 2005. This conclusion is consistent with the findings that runoff in the upper reaches of the HRB is characterized by high flow due to the warm-wet climate [17–18]. Thus, the surface water resources from the Yingluoxia hydrological station flowing into the middle reaches of the basin have slightly increased.

Nevertheless, the available surface water resources observed in the middle reaches over 2000–2011 decreased compared with the 1990s. The amount is equal to the difference in the water allocation between the Yingluoxia and Zhengyixia hydrological stations (Fig 3) and is approximately equal to the total water consumption of the middle reaches. From 1990 to 1999, the average available surface water was 8.10 × 10^8^ m^3^. However, during 2000 and 2011, the average available surface water was reduced to 7.55 × 10^8^ m^3^, a decrease of 0.55 × 10^8^ m^3^. Furthermore, the available surface water significantly decreased between 2000 and 2006. Therefore, the available surface water was limited between 2000 and 2011, although a significant increase in the water from the Yingluoxia hydrological station flowed into the middle reaches. The implementation of the EWDP in 2000 controlled the amount of water diversion in the middle reaches. Simultaneously, the total area of farmland increased by 12.01% (256.14 km^2^) between 2000 and 2011. Based on an irrigation quota of 4,856 m^3^/acre, the new farmland required approximately 3.07 × 10^8^ m^3^ of water for irrigation. However, the surface water for irrigated farmland decreased by 1.25 × 10^8^ m^3^ from 2000 to 2010 in Ganzhou District, Linze County and Gaotai County [48]. The surface water resources were therefore insufficient.

Groundwater was extracted to irrigate the expanded farmland in case of a surface water shortage. In the field survey, we found that most of the newly reclaimed farmland only relied on groundwater irrigation. According to statistics provided by the Zhangye Water Authority, the number of agricultural motor-pumped wells increased from 5,547 in 2000 to 9,297 in 2010 in Ganzhou District, Linze County and Gaotai County. The amount of mined groundwater for irrigated farmland increased by 1.64 × 10^8^ m^3^ [48]. Therefore, the groundwater was overexploited.

### Driving factors

**Fig 4:**
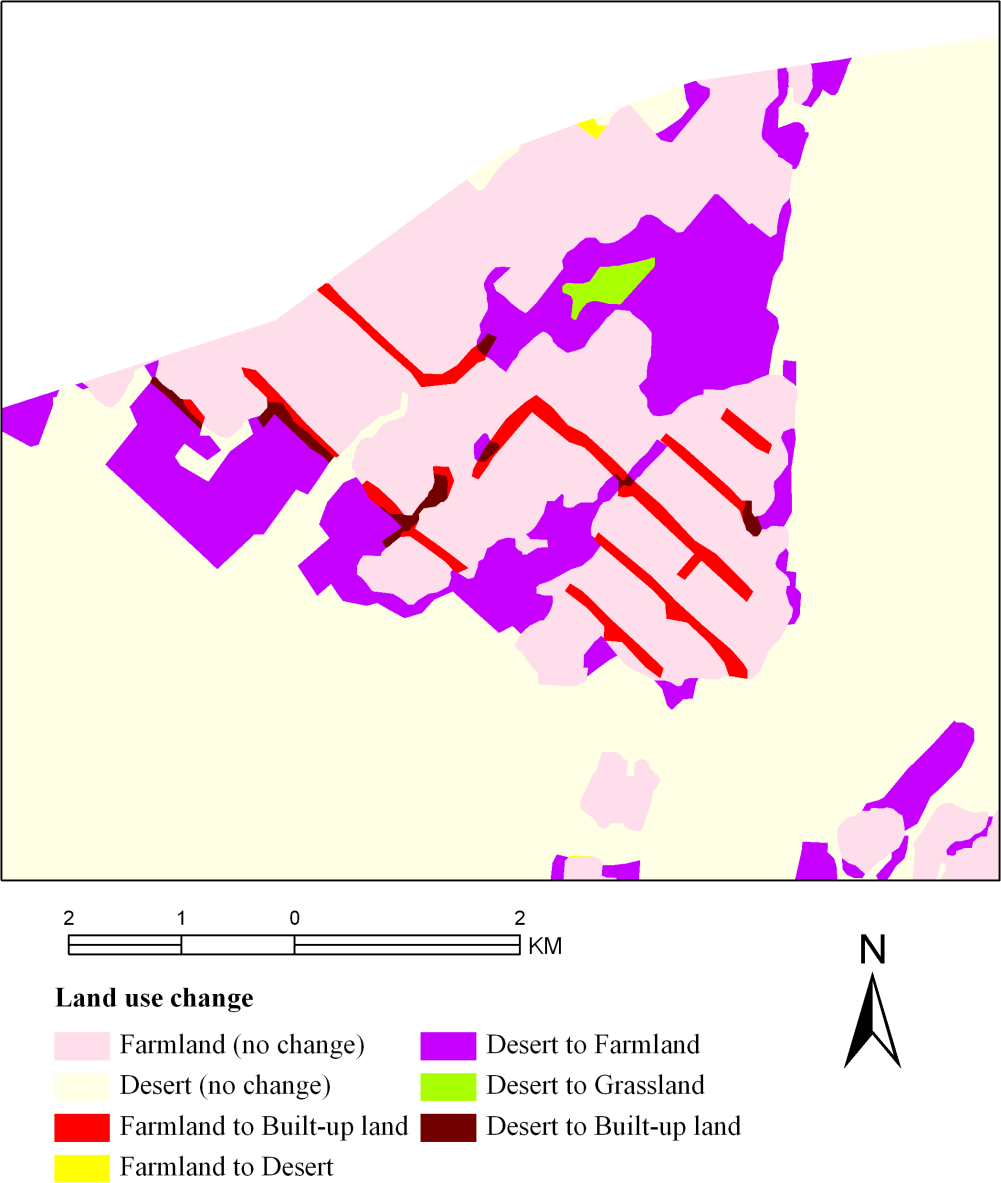
Case 1: farmland area changes in Xusanwan, Gaotai County, from 2000 to 2007, driven by migrants.

**Fig 5:**
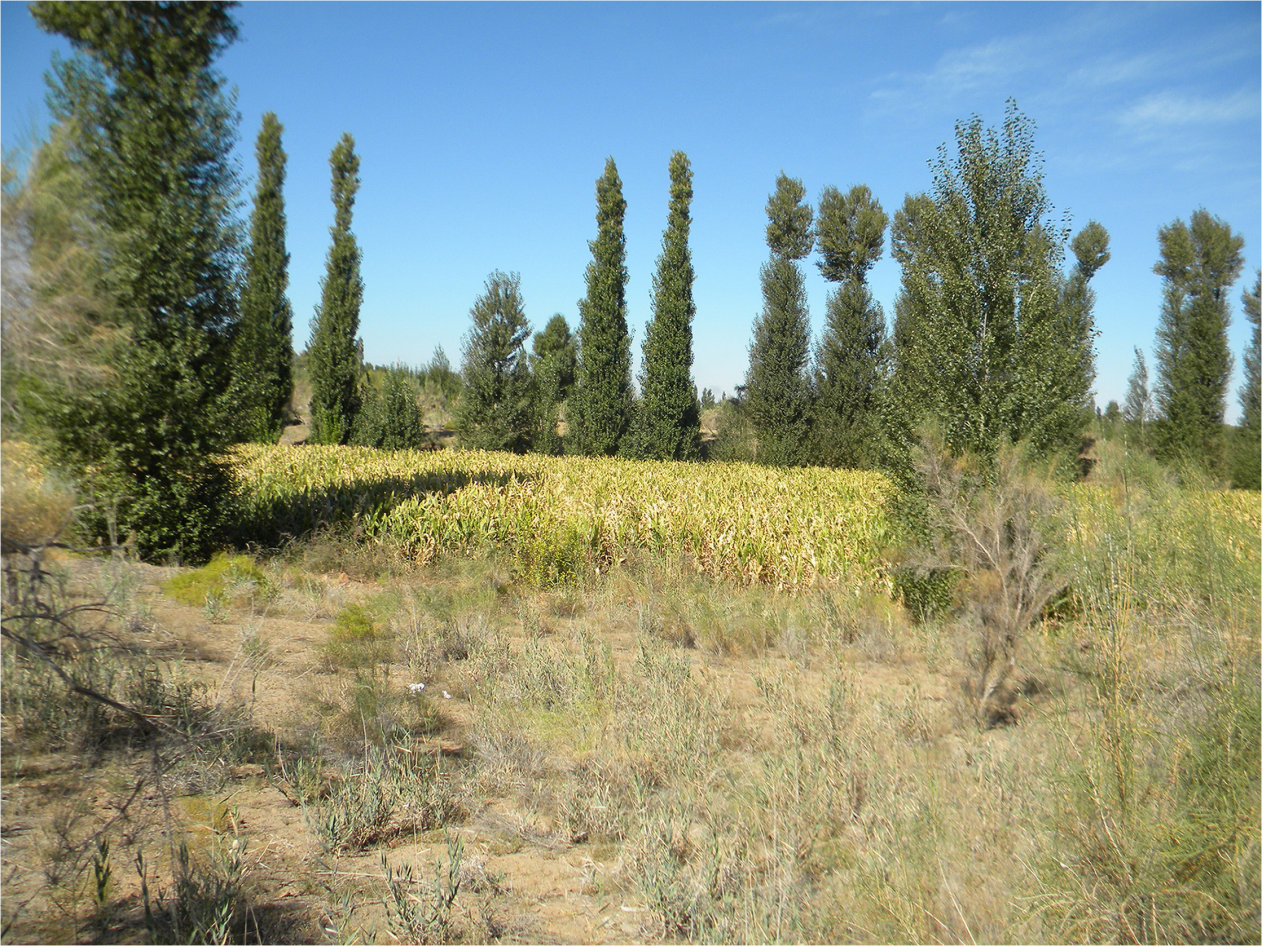
Newly reclaimed farmland in the inter-dune zone.

Land use/cover change is often correlated with the implementation of national policy [49], which has been considered a major factor of land use/cover change [50–51]. In the framework of the EWDP, the ecological migration and ‘grain-for-green’ policies were implemented in the middle reaches of the HRB. The impact of these polices on the expansion and abandonment of farmland is obvious. In the middle reaches of the HRB, the regional expansion of farmland was at least partly driven by migrants. Figs 2 and 4 show an example of this phenomenon. Farmland reclamation was very common in Xusanwan, Gaotai County because of the large number of immigrants from the piedmont irrigation district. As a result, the total area of farmland in this region increased by 45% between 2000 and 2007. The ‘grain-for-green’ policy also had an effect on the proportion of the landscape covered by farmland. The main feature of this program provided free grain and cash payments to participating farmers who converted farmland to forests and grassland [52]. After the implementation of the ‘grain-for-green’ policy, arboreal forests, shrub forests, thick grasslands and moderate grasslands in the middle reaches of the HRB increased, albeit by varying degrees (7.76%, 12.78%, 21.88% and 2.91%, respectively), between 2000 and 2007. However, in recent years, some farmers who converted farmland to grassland were not compensated with grain or cash. This outcome, and the price increase for agricultural products, has led farmers to plant crops on farmland that had been converted to grassland or to reclaim new farmland. In the field survey, we also observed that much desert, sparse grassland, sparse forest and overflow land had been reclaimed as farmland by local people (Fig 5).

Another important result of policy implications was wetland change. From 2000 to 2007, the wetland area decreased. However, after the implementation of the Wetland Conservation Project, the wetland area began to increase, with a greater increase in partial wetland areas. For example, in the northern area of Ganzhou District, the wetland area increased by 5.64 km^2^ between 2007 and 2011, which is equivalent to an increase of 61.86% in the total wetland area in the same period.

**Fig 6:**
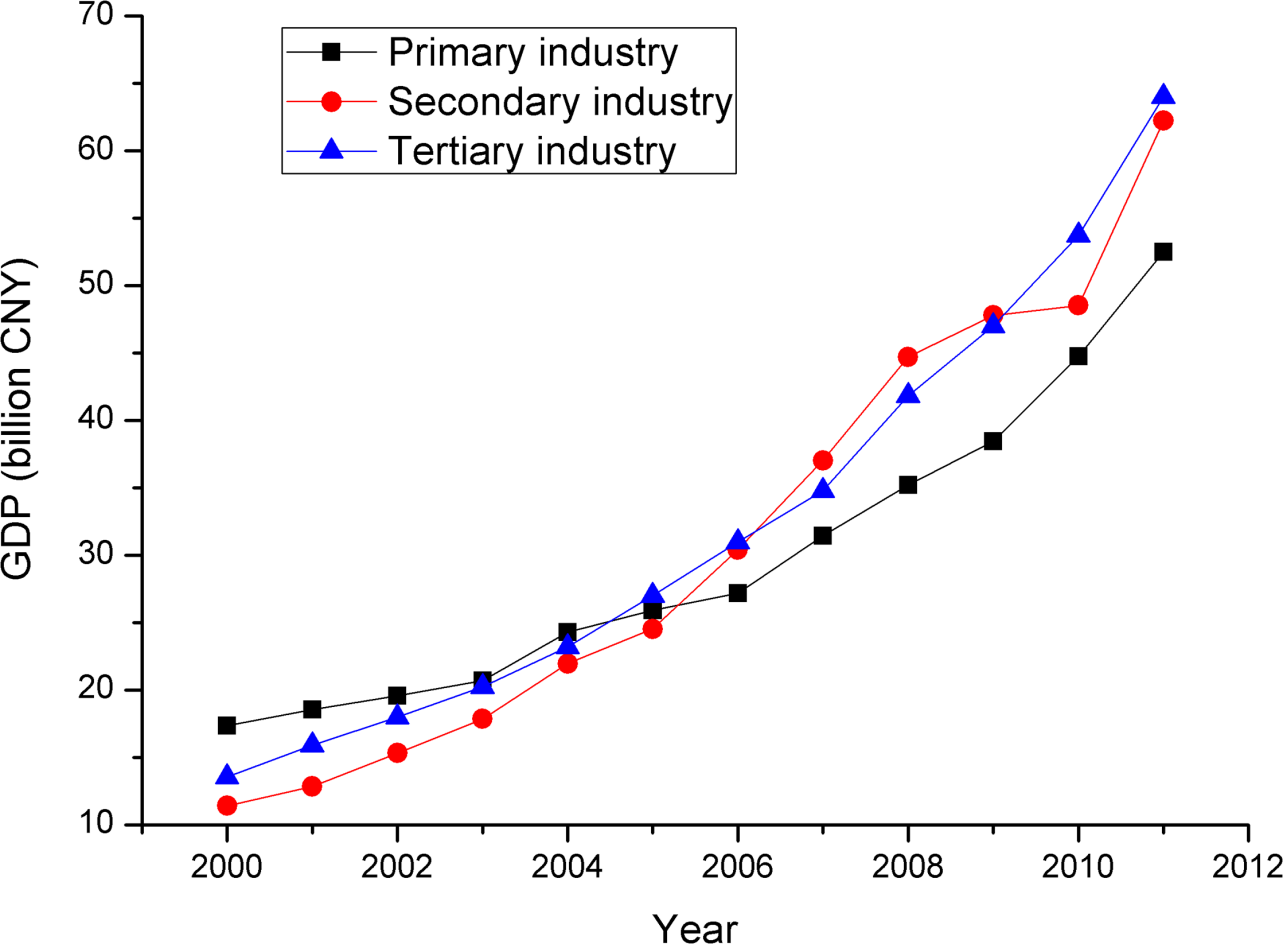
GDP change during 2000–2011.

The above analysis and the results of the field survey indicate that the implementation of nation policy only affected the evolution of patterns of land use/cover in particular areas of the middle reaches. Outweighing national policy, the strong willingness to pursue economic interests significantly affected the land use/cover change. The expansion of farmland was particularly driven by this factor. After 2000, socioeconomic development proceeded very rapidly in the middle reaches of the HRB. In 2000, the GDP was 42.32 billion CNY. In 2011, the GDP reached 178.71 billion CNY. The average annual growth rate of the GDP between 2000 and 2011 was approximately 13.99%. The average annual growth rates of primary, secondary and tertiary industries over the same period were 10.62%, 16.67% and 15.13%, respectively (Fig 6). The use of expanded farmland to grow crops with high economic returns, such as seed corn, significantly increased income. In 2011, the income of the corn seed industry reached 24 billion CNY. The per capita income of a farmer from seed corn was 1800 CNY, which accounted for 32% of the average farmer’s per capita income [53]. In the middle reaches, farmland increased by 256.14 km^2^ between 2000 and 2011, primarily because of seed corn, which has a high economic return. The pursuit of economic interests was therefore a significant factor in the farmland expansion in the middle reaches of the basin. This finding was similar to the research results of land use/cover change in the other arid areas of northwestern China, such as Gansu [54], Qinghai [55] and Xinjiang [56].

### Consequences for the ecosystem

**Fig 7:**
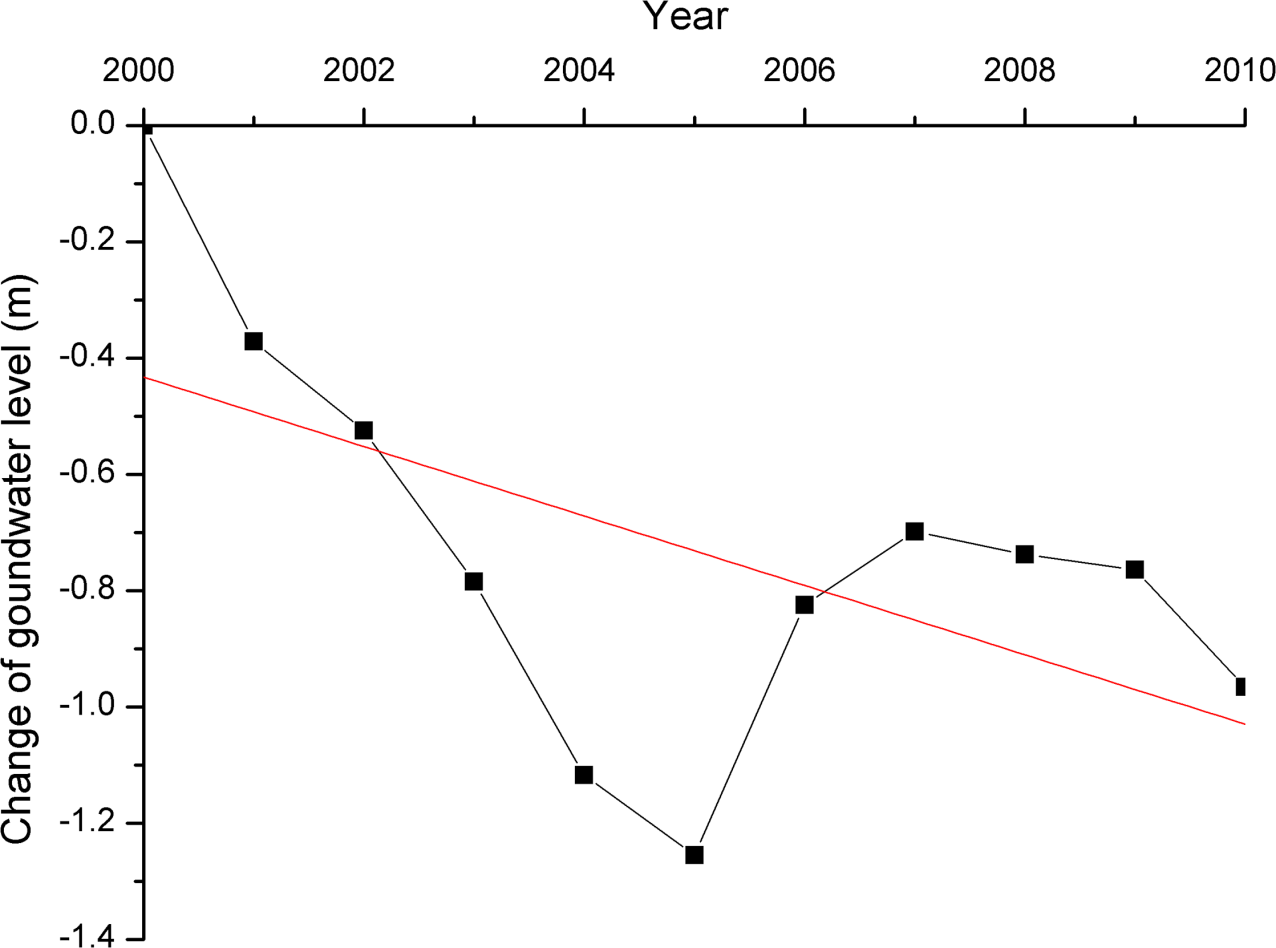
Average change in the groundwater level at 42 observation wells in the middle reaches of the HRB. The red line indicates trends over the past ten years.

The middle reaches of the HRB are a water-scarce region, where the available surface water resources are limited. Of the available surface water resources, approximately 90% was used for irrigation, whereas only 6% was used for natural ecosystems. Strong competition for water occurs between irrigated farmland and natural ecosystems in the region [22]. A substantial increase in the farmland inevitably led to the over-extraction of groundwater and a transfer of water from ecosystems. The over-extraction of groundwater has resulted in declining groundwater levels in particular areas of the middle reaches. A groundwater-level analysis of 42 observation wells in the middle reaches of the HRB revealed that the groundwater levels generally decreased between 2000 and 2010 (Fig 7). Additionally, water use data from various sectors showed that the amount of ecological water decreased from 1.76 × 10^8^ m^3^ in 2000 to 1.14 × 10^8^ m^3^ in 2010, according to the statistics provided by the Zhangye Water Conservancy Annual Report. The declining groundwater levels and the reduction in ecological water led to the reduction of shrub forests and an increase in grassland desertification in particular areas of the middle reaches. In the field survey, we found that a large area of *Elaeagnus* forest exhibited drought stress or partial die-out in the tree farms of Jiulong Jiang. This effect was probably due to declining groundwater levels and ecological water. In addition, the large-scale reclamation of farmland and the reduction of available surface water resulted in other changes in land use/cover types, such as a drastic decrease in the grassland area. From 2000 to 2011, the grassland area decreased by 6.26%.

### Implications for water and land management

Based on the discussion above, the natural ecosystem has been degraded and the groundwater has declined in particular areas of the middle reaches of the HRB basin. However, these problems are manageable because the runoff from mountainous areas is currently high. Nevertheless, the expansion of farmland areas in the middle reaches will present a severe challenge for local governments when continuous low-flow years occur. This trend may also threaten the sustainability of the entire river basin. Farmland area should thus be strictly controlled. We recommend that farmland in the transition zone between the oasis and desert be gradually abandoned to control the farmland area. Farmland abandonment can be implemented in two phases over ten years. An increase in income for farmers who abandon farmland is crucial. Therefore, a rational ecological compensation system for farmland abandonment should be established. Compensation standards should be proposed according to the current economic conditions. Simultaneously, the transfer program for rural laborers who may lose their land should be revised and improved to create more employment opportunities. In addition to the development of industry and tourism, the local government can focus on the development of the rural service industry according to the local characteristics. Local governments should further strengthen the management of water resources, particularly the management of groundwater. Groundwater exploitation should be strictly controlled to successfully limit the farmland area and to achieve sustainable water use. In summary, appropriate measures toward the sustainable use of water and land include controlling the farmland area and strengthening the water resource management.

## Conclusions

We investigated changes in land use/cover in the middle reaches of the HRB between 2000 and 2011, discussed the driving factors of these changes, and determined the implications for sustainable water resource management. Land use/cover in the middle reaches of the HRB has changed significantly over this period. Areas of farmland and built-up land significantly increased. In contrast, the areas occupied by grasslands, water bodies and deserts continuously decreased during 2000–2011. Wetland areas first decreased by 5.78% between 2000 and 2007 and then increased by 5.46% between 2007 and 2011.

The most significant land use/cover change in the middle reaches of the HRB over 2000–2011 was the continuous expansion of farmland, which mainly resulted from economic interests. From 2000 to 2011, the farmland area increased by 12.01%. The expansion of farmland increased the water resource stress, resulting in the over-extraction of groundwater and ecosystem degradation in particular areas. Both consequences are negative and are potential threats to the sustainability of the middle reaches of the HRB and the entire river basin.

We suggest that a compromise between economic benefits and sustainable water use is needed. Groundwater should not be over-extracted, and the environmental water flow should be increased. To achieve these goals, the farmland area should be strictly controlled. Unauthorized reclamation should not be allowed, and if possible, some farmland in the transition zone between the oasis and desert should be restored to natural vegetation. Therefore, the river basin sustainability could be guaranteed. Overall, local governments should improve the management of water resources, particularly the management of groundwater, and should strictly control the reclamation of farmland. As a result, the ecologically and socioeconomically sustainable use of water resources and a balance between upstream and downstream water demands could be achieved.

The results of this study suggest that the rational allocation of water and land resources in this arid inland river basin and coordinated ecological and economic development are possible. These results can be regarded as a reference to guide the sustainable management of water resources in other arid inland river basins.

## References

[1] Lambin EF, Turner BL, Geist HJ, Agbola SB, Angelsen A, Bruce JW, et al The cause of land-use and land-cover change:moving beyond the myths. Global Environmental change. 2001; 11(4): 261–269.

[2] Giri C, Pengra B, Long J, Loveland TR. Next generation of global land cover characterization, mapping, and monitoring. International Journal of Applied Earth Observation and Geoinformation. 2013; 25: 30–37.

[3] Chase TN, Pielke RA, Kittel TGF, Nemani RR, Running SW. Simulated impacts of historical land cover changes on global climate in northern winter. Climate Dynamics. 1999; 16: 93–105.

[4] DeFries R, Eshleman KN. Land-use change and hydrologic processes: a major focus for the future. Hydrological Process. 2004; 18: 2183–2186.

[5] Wang GX, Liu JQ, Kubota J, Cheng L. Effects of land-use changes on hydrological processes in the middle basin of the Heihe River, northwest China. Hydrological Processes. 2007; 21: 1370–1382.

[6] Foley JA, DeFries R, Asner GP, Barford C, Bonan G, Carpenter SR, et al Global consequences of land use. Science. 2005; 309: 570–574. 16040698 10.1126/science.1111772

[7] Turner BL, Lambin EF, Reenberg A. The emergence of land change science for global environmental change and sustainability. Proceedings of the National Academy of Sciences. 2007; 104(52): 20666–20671. 10.1073/pnas.0704119104 PMC2409212 18093934

[8] Luo GP, Zhou CH, Chen X, Li Y. A methodology of characterizing status and trend of land changes in oases: A case study of Sangong River watershed, Xinjiang, China. Journal of Environmental Management. 2008; 88: 775–783. 17531372 10.1016/j.jenvman.2007.04.003

[9] Cai XM, McKinney DC, Rosegrant MW. Sustainability analysis for irrigation water management in the Aral Sea region. Agricultural systems. 2003; 76(3): 1043–1066.

[10] Fang CL, Bao C, Huang JC. Management implications to water resources constraint force on socio-economic system in rapid urbanization: a case study of the Hexi Corridor, NW China. Water Resources Management. 2007; 21: 1613–1633.

[11] Cheng GD. Saving water is the only way for Northwest China to survive. Bulletin of the Chinese Academy of Sciences. 1996; 10(3): 203–206.

[12] Cheng GD, Li X, Zhao WZ, Xu ZM, Feng Q, Xiao SC, et al Integrated study of the water-ecosystem-economy in the Heihe River Basin. National Science Review. 2014; 1(3): 413–428.

[13] Xiao SC, Xiao HL. The impact of human activity on the water environment of Heihe water basin in last century. Journal of Arid Land Resources and Environment. 2004; 18(3): 57–62.

[14] Wang J, Meng JJ. Characteristics and tendencies of annual runoff variations in the Heihe River Basin during the past 60 years. Scientia Geographic Sinica. 2008; 28(1): 83–88.

[15] Yang B, Qin C, Shi F, Sonechkin DM. Tree ring-based annual streamflow reconstruction for the Heihe River in arid northwestern China from AD 575 and its implications for water resource management. The Holocene. 2011; 22(7): 773–784.

[16] Kang XC, Cheng GD, Kang ES, Zhang QH. The runoff reconstruction of Heihe River Basin using tree rings in the recent thousand years. Science in China (Series D). 2002; 32: 675–85.

[17] Shi YF, Shen YP, Li DL, Zhang GW, Ding YJ, Hu RJ, et al Discussion on the present climate change from warm-dry to warm-wet in northwest China. Quateranry Sciences. 2003; 23(2): 152–164.

[18] Qin C, Yang B, Burchardt I, Hu XL, Kang XC. Intensified pluvial conditions during the twentieth century in the inland Heihe River. Global and Planetary change. 2010; 72: 192–200.

[19] Wang GX, Cheng GD. Water resource development and its influence on the environment in arid areas of China—the case of the Hei River basin. Journal of Arid Environments. 1999; 43(2): 121–131.

[20] Li X, Lu L, Cheng GD, Xiao HL. Quantifying landscape structure of the Heihe River Basin, northwest China using FRAGSTATS. Journal of Arid Environments. 2001; 48(4): 521–535.

[21] Cheng GD, Wang GX. Changing trend of drought and drought disaster in northwest China and countermeasures. Earth Science Frontier. 2006; 13(1): 3–14.

[22] Lu L, Li X, Veroustraete F, Kang E, Wang J. Analysing the forcing mechanisms for net primary productivity changes in the Heihe River Basin, north-west China. International Journal of Remote Sensing. 2009; 30(3): 793–816.

[23] Gao QZ, Wu YQ. Analysis of water cycle in inland river basins in Hexi region. Advances in Water Science. 2004; 15(3): 391–396.

[24] Ji XB, Kang ES, Chen RS, Zhao WZ, Zhang ZH, Jin BW. The impact of the development of water resources on environment in arid inland river basins of Hexi region, Northwestern China. Environmental Geology. 2006; 50: 793–801.

[25] Liu H, Cai XM, Geng LH, Zhong HP. Restoration of pastureland ecosystems: case study of Western Inner Mongolia. Journal of Water Resources Planning and Management. 2005; 131(6): 420–430.

[26] Liu YC. Evolution of the Juyan lake. Journal of Arid land Resources and Environment. 1992; 6(2): 9–18.

[27] Feng ZM, Yang YZ, Zhang YQ, Zhang PT, Li YQ. Grain-for-green policy and its impacts on grain supply in West China. Land Use Policy. 2005; 22: 301–312.

[28] Wang XH, Lu CH, Fang JF, Shen YC. Implications for development of grain-for-green policy based on cropland suitability evaluation in desertification-affected north China. Land Use Policy. 2007; 24: 417–424.

[29] Zhang YC, Yu JJ, Wang P, Fu GB. Vegetation responses to integrated water mangement in the Ejina basin, northwest china. Hydrological Processes. 2011; 25: 3448–3461.

[30] Chang YS, Bao D, Bao YH. Satellite monitoring of the ecological environment recovery effect in the Heihe River downstream region for the last 11 year. Procedia Environmental Sciences. 2011; 10: 2385–2392.

[31] Li N, Yang TB. Research on LUCC in Middle Reaches of Heihe River Basin after Water Reallocation. Journal of Desert Research. 2008; 28(2): 223–226.

[32] Wei Z, Jing HJ, Lan YC, Wu JK, Hu XL, Ji YJ. Study on the change of groundwater level and reserves in the Lower Reaches of the Heihe River after redistributing water. Arid Zone Research. 2008; 25(3): 336–341.

[33] Lu L, Li X, Cheng GD. Landscape evolution in the middle Heihe River Basin of northwest China during the last decade. Journal of Arid Environments. 2003; 53(3): 395–408.

[34] Meng JJ, Wu XQ, Li ZG. Land use and land cover changes in Heihe River Basin during the period of 1988–2000. Acta Scientiarum Naturalium Universitatis Pekinensis. 2004; 40(6): 922–929.

[35] Wang GX, Liu JQ, Chen L. Comparison of spatial diversity of land use changes and the impacts on two typical areas of Heihe River Basin. Acta Geographica Sinica. 2006; 61(4): 339–348.

[36] Li CZ, Yu FL, Liu J, Yan DH, Zhou T. Research on land use/cover change and its driving force in midstream of the Heihe mainstrem basin during the past 20 years. Journal of Natural Resources. 2011; 26(3): 354–361.

[37] Kilgour DM, Dinar A. Flexible water sharing within an international river basin. Environmental and Resource Economic. 2001; 18: 43–60.

[38] Micklin PP. Desiccation of the Aral Sea: a water management disaster in the Soviet Union. Science. 1988; 241(4870): 1170–1176. 17740781 10.1126/science.241.4870.1170

[39] Micklin P. The Aral sea disaster. Annu. Rev. Earth Planet. Science. 2007; 35: 47–72.

[40] Deng MJ, Long AH. Evolution of Hydrologic and Water Resources and Ecological Crisis in the Aral Sea Basin. Journal of Glaciology and Geocryology. 2011; 33(6): 1363–1375.

[41] Feng Q, Liu W, Si JH, Su YH, Zhang YW, Cang ZQ, et al Environmental effects of water resource development and use in the Tarim River basin of northwestern China. Environmental Geology. 2005; 48(2): 202–210.

[42] Zhang B, Zhang K. Analysis of industrial structure situation according to water resources in arid area–a case study of the middle reaches of Heihe River Valley. Areal research and development. 2004; 23(5): 112–115.

[43] Liu JY, Liu ML, Zhuang DF, Zhang ZX, Deng XZ. Study on spatial pattern of land-use change in China during 1995–2000. Science in China Series D. 2003; 46: 373–384.

[44] Liu JY, Liu ML, Tian HQ, Zhuang DF, Zhang ZX, Zhang W, et al Spatial and temporal patterns of China’s cropland during 1990–2000: An analysis based on Landsat TM data. Remote sensing of Environment. 2005; 98: 442–456.

[45] Kloditz C, Boxtel AV, Carfagna E, Deursen WV. Estimating the accuracy of coarse scale classification using high scale information. Photogrammetric engineering and remote sensing. 1998; 64(2): 127–132.

[46] Ran YH, Li X, Lu L. Evaluation of four remote sensing based land cover products over China. International Journal of Remote Sensing. 2010; 31(2): 391–401.

[47] Li X, Nan ZT, Cheng GD, Ding YJ, Wu LZ, Wang LX, et al Toward an improved data stewardship and service for environmental and ecological science data in west China. International Journal of Digital Earth. 2011; 4(4): 347–359.

[48] Ge YC, Li X, Tian W, Zhang YL, Wang WZ, Hu XL. The impacts of water delivry on artificial hydrological circulation system of the middle reaches of the Heihe River Basin. Advances in Earth Science. 2014; 29(2): 285–294.

[49] Zhang JX, Liu ZJ, Sun XX. Changing landscape in the Three Gorges Reservoir Area of Yangtze River from 1977 to 2005: Land use/land cover, vegetation cover chagnes estimated using multi-source satellite data. International Journal of applied earth observation and geoinformation. 2009; 11(6): 403–412.

[50] Yang HF, Mu SJ, Li JL. Effects of ecological restoration projects on land use and land cover change and its influences on territorial NPP in Xinjiang, China. Catena. 2014; 115: 85–95.

[51] Zhou DC, Zhao SQ, Zhu C. The Grain for Green Project induced land cover change in the Loess Plateau: A case study with Ansai County, Shanxi Province, China. Ecological indicators. 2012; 23: 88–94.

[52] Long HL, Heilig GK, Wang J, Li XB, Luo M, Wu XQ, et al Land use and soil erosion in the upper reaches of the Yangtze River: some socio-economic considerations on China’s Grain-for-green Programme. Land Degradation & Development. 2006; 17: 589–603.

[53] Song XL. Study on the development of corn seed industry in Zhangye city. China seed industry. 2013; 1: 27–29.

[54] Xie YC, Gong J, Sun P, Gou XH. Oasis dynamics change and its influence on landscape pattern on Jinta oasis in arid China from 1963a to 2010a: Integration of multi-source satellite images. International Journal of applied earth observation and geoinformation. 2014; 33: 181–191.

[55] Wang XH, Zheng D, Shen YC. Land use change and its driving forces on the Tibetan Plateau during 1990–2000. Catena. 2008; 72: 56–66.

[56] Zhou DC, Luo GP, Lu L. Processes and trends of the land use change in Aksu watershed in the central Asia from 1960 to 2008. Journal of arid land. 2010; 2: 157–166.

